# Breaking Taboos: Arab Breast Cancer Activism in Art and Popular Culture

**DOI:** 10.1007/s10912-024-09886-6

**Published:** 2024-09-13

**Authors:** Abir Hamdar

**Affiliations:** School of Modern Languages and Cultures, Durham University, New Elvet, Elvet Riverside, Durham, DH1 3JT UK

**Keywords:** Arab, Cancer, Activism, Popular culture, Art

## Abstract

This essay examines the breast cancer accounts of four Arab female celebrities who have spoken out in public about their illness experience: the Egyptian TV presenter Basma Wahba and the actress Yasmine Ghaith, the Iraqi actress Namaa al-Ward, and the Lebanese pop singer Elissa. By reading their testimonies against the backdrop of critical literature on illness narratives and memoirs, as well as on cancer narratives and activism, the essay asks: how are the accounts of these women’s cancer diagnosis and treatment disclosed and described? In what medium do they communicate and circulate their breast cancer experiences? What significance do these public disclosures have on challenging and breaking the Arab taboo of cancer? In conclusion, the essay argues that these women’s willingness to share their stories in public constitutes an important form of multimedia activist intervention—visual, sonic, and performative—that is playing a key role in the development of a breast cancer movement.

## Introduction

In an interview,^1^ Saudi Arabian obstetrician and gynecologist Samia al-Amoudi^2^ recalls her diagnosis of breast cancer in 2006 and says that the first thing her family and friends told her upon learning the news was “Now that you’ve got breast cancer, let’s keep it between ourselves. There’s no need for people to know that you’ve got breast cancer.” She adds: “That was shocking to me, because I felt that they were taking it as if it was something wrong, a taboo that we should hide (…)” (qtd. in Damanhouri 2015). Al-Amoudi, who was diagnosed with cancer twice and has since written about her own experience to raise awareness and to help break the silence, further observes:

The main problem that women confront in the Arab culture (…) is the taboo about discussing medical issues that involve an intimate female body part. So, if women can’t talk about breast cancer, it prevents them from accessing regular early detection tests to catch the cancer at an early, curable stage. (qtd. in Piana 2014)

To be sure, like many other societies across the globe, cancer was for a long time a taboo in Arab society. As I have argued elsewhere, this taboo status is best captured by the fact that the disease has taken on different, and euphemistic, names in Arabic: *Haydek al-Marad* [that disease], *Haydek al-Shi* [that thing], *Al-Marad al-Wihish* [the bad or nasty disease], *Al-Marad al- ‘Atel* [the dreadful or lousy disease], and *Al-Marad illi Mabyitsama* [the disease not to be named] among others. Even when the disease is named, it is usually in a foreign language such as “cancer” or “cancre” and is seldom identified by its Arabic medical name “*saratan*” (Hamdar 2021, 197). When cancer is the subject of conversation, phrases such as “may God keep it at bay” and “may God save us from it” are often uttered with intense feeling. If it is possible to speak of a broader “cultural shift” in the ways in which the West refers to and represents cancer (DeShazer 2010, 2), to the point where it has become “the stuff of daily discourse” (Burke 2013, xxx), it thus bears stressing that this shift has not taken place in the Arab world. Despite specific socio-cultural contexts across different parts of the Arab world, cancer continues to carry taboo connotations while the experience of cancer is often lived in silence. This silence surrounding cancer is particularly the case for Arab women diagnosed with the disease.

In the past few years, however, we have begun to witness a number of attempts at disrupting this culture of silence and shame where Arab female cancer experiences are concerned. To quickly summarize, this effort has been largely carried out by a number of Arab female public figures and artists who have spoken about their cancer experiences—in particular breast cancer—on different media platforms and through a number of Arab cancer organizations^3^ including the late Palestinian singer Rim Banna,^4^ the Jordanian singer and actress Amal Dabbas, the Kuwaiti actress Zahra al-Kharji, the Lebanese journalist and writer Hoda Chedid, and the Lebanese-Syrian singer Nora Rahal. While this list is not exhaustive, it, nevertheless, gives an indication of the extent to which some Arab women who are often visible in the public sphere have invested in the struggle against the taboo of cancer—and breast cancer more specifically—by drawing on their own experiences. This essay examines the breast cancer accounts of four Arab female celebrities who have spoken out in public about their illness experience: the Egyptian TV presenter Basma Wahba and the actress Yasmine Ghaith, the Iraqi actress Namaa al-Ward, and the Lebanese pop singer Elissa. By reading their testimonies against the backdrop of critical literature on illness narratives and memoirs, as well as on cancer narratives and activism, the essay asks: how are the accounts of these women’s cancer diagnosis and treatment disclosed and described? In what medium do they communicate and circulate their breast cancer experiences? What significance do these public disclosures have on challenging and breaking the Arab taboo of cancer? In conclusion, this essay argues that these women’s willingness to share their stories in public constitutes an important form of multimedia activist intervention—visual, sonic, and performative—that is playing a key role in the development of a breast cancer movement.

## Breast cancer activism

In their analysis of cancer awareness campaigns in Western contexts, Johansen et al. posit that “few other diseases – if any – have received as much public attention as breast cancer” (2013, 141). The concentrated effort to raise awareness of breast cancer is, of course, in sharp contrast to the history of silence that has marked cancer experiences more generally and breast cancer more specifically. For Ellen Leopold, breast cancer has “with few exceptions, (…) been hidden away since time immemorial” (2006, 60):

The taboo against drawing public attention to it was firmly in place. Where the subject did surface in print (in medical texts or health manuals for women), the disease was often traced to “inappropriate” behaviour on the part of the hapless female victim—too much dancing, exercising, or thinking often were cited as causes. (62)

If James Olson also cites examples from as late as the 1950s whereby “the stigma of losing a breast” led many women to “swear their physicians and nurses” into silence (2002, 101), Stacey (1997) and Potts (2000) have shown that this culture of silence towards breast cancer began to change by the 1960s. In the United States, for example, a number of well-known American women made their breast cancer prognoses public, paving the way for other women to speak out about the disease. These breast cancer stories were initially published in books, newspapers, and magazines and then told on radio and television (Stacey 1997; Potts 2000; Olson 2002; Leopold 2006; Pitts 2004; Orgad 2005).

Coupled with all this was the extensive work of grassroot organizations, some of which were founded in the United States but now operate globally: the Susan G. Komen Breast Cancer Foundation launched in 1982, for example, whereas the Pink Ribbon came into existence in 1992. Charities, health organizations, and even the fashion and entertainment industries have also all contributed to activities to raise awareness of breast cancer (Johansen et al. 2013, 141). Today, breast cancer activism has “become a fixture” in many countries, including in the United States and Greater Britain where breast cancer activists have “earned a role in determining research and funding priorities” (Radley et al. 2007, 366). Consequently, Western attitudes and perceptions of breast cancer have shifted dramatically. The twenty-first century has also seen breast cancer activists address “previously neglected topics” such as environmental risks, genetic testing, preventive mastectomy, and corporate philanthropy, thus engaging the public “politically, ethically and aesthetically” (DeShazer 2013, 1–2). In this context, we encounter the ways in which, according to Maren Klawiter, the very “disease regime” of breast cancer—which is to say “institutionalised practices, authoritative discourses, social relations, collective identities, emotional vocabularies, visual images, public policies and regulatory actions through which diseases are socially constituted and experienced” (2004, 845, 851)—has radically shifted.

However, this dramatic transformation in how cancer, and breast cancer especially, is perceived, represented, and narrated in North America and Europe is in sharp contrast to Arab contexts where public attitudes towards the disease remain affected by the cancer taboo. To recall Salwa Saadeh and Hikmat Abdel-Razeq’s verdict in a 2022 essay on breast cancer in the Arab world:

Unfortunately, even in this time and age, cancer in general and breast cancer, in particular, is associated with significant social stigmatization in many countries and societies in the Arab world, regardless of socio-economic and educational development in these countries. Women diagnosed with breast cancer often describe a feeling of shame or guilt, and fear of being blamed for having cancer, as at times, it is regarded as a sign of punishment for undisclosed sins. (357)

So, any attempt to tell the story of Arab breast cancer activism must start with the acknowledgement that it is largely in its nascent years, despite the variation in activities and progress across different Arab contexts. It is true that in the past decade or so we have seen the establishment of various breast cancer foundations, charities, and support groups across the Arab world that have made concentrated attempts to raise awareness of early breast cancer screening, detection, and treatment,^5^ nevertheless, the social stigma and taboo of cancer, and breast cancer in particular, persists. In the words of leading breast cancer specialist Lebanese Naji el-Saghir of the American University of Beirut Medical Center: “The recent explosion of knowledge and information through the media and internet have helped change some attitudes, but most people still refer to it as ‘that other disease’ and remain afraid of mentioning it by name” (2007, 226).

Even more troubling is that in a region where physicians have noted that, on average, breast cancer occurs at an earlier age and at a more advanced stage than industrialized countries (Najjar et al., 2010; Ezzat et al. 1999), women still find it difficult to seek “higher indices of suspicion and early diagnosis” due to a number of factors including “fear of cancer [and] shyness” (el-Saghir et al. 2007, 229). Speaking in the context of Saudi Arabia, for example, Modia Batterjee—Co-Founder of Al-Bidayah Breastfeeding & Women’s Health Organization—notes that many women are reluctant to undergo self-examination or to teach their daughters to examine their breasts: “On many occasions I have been approached by women telling me they do not want their girls to learn how to touch themselves.” Similarly, clinical psychologist and author of *Riyadh-London: Something in my Chest*, Omaymah al-Tamimi, adds that “Our culture restricts women from having a more aware relationship with their bodies, so they wouldn’t know how their breasts would feel prior to the presence of a prominent lump” (qtd. in Shiraz 2015).

In many ways, the perception of cancer as an end-of life journey along with persistent conservative attitudes towards women’s bodies in illness and health (Hamdar 2014; Khattab 1992) have meant that not only do Arab women delay screening examinations or even refuse to undergo them, they also “hide away and some even refuse treatment” because they “believe they are socially unaccepted” (qtd. in Kinias 2017). Despite or because of these overriding attitudes, the past few years have brought about the beginning of what may prove to be a paradigm shift insofar as breast cancer experiences are concerned. This shift is not solely the result of awareness campaigns run by breast cancer foundations and groups, but rather the effect of women who have or had experienced cancer and who are emerging, to borrow Klawiter’s words, “from the architecture of the closet and reconstitut[ing] themselves as political actors’ (2008, xxii). In what follows, I focus on the breast cancer experiences and “coming out” narratives of women who are in the public sphere and whose accounts of their illness experience has, on multiple occasions, broken several taboos associated with the disease. If the women featured in this essay can be seen as middle-class, educated, and/or possessing celebrity status, then it is worth stressing that despite this inherent tension in their privileged position, they remain engaged in a grassroots awareness raising agenda and their experiences as well as its mode of transmission connects with and is marked by its engagement with women of all walks of life.

## Basma Wahba and media accounts

In July 2013, during the Muslim fasting month of Ramadan, Egyptian female journalist and TV presenter Basma Wahba appeared on the TV talk-show *Ana wal- ‘Asal* [Me and Honey], which is hosted by the well-known Lebanese presenter Neshan Der Haroutiounian (widely known as Neshan), to discuss Wahba’s experience with breast cancer and her subsequent mastectomy. To start with, Wahba’s decision to openly discuss her cancer diagnosis and treatment on a popular television show during a period which historically attracts very large viewing audiences broke a significant taboo. If the episode was clearly intended to inspire, the content and language of the interview was also controversial for many because it appeared to reinforce, rather than challenge, existing perspectives and attitudes towards the disease. For example, Neshan opened the interview by asking his guest in a dramatic or even melodramatic voice: “Tell me directly, how could such a disease – cancer – come to [a woman of] such beauty, such high spirits and such strength and will?” to which Wahba responded by reminding Neshan of the “demonic” powers of cancer: “Of course, it is called the ‘evil disease’ and the ‘demonic disease,’ and I had to be strong (…) It invaded my body and I had no option but to get up and face it. It was either it or me” (*Ana wal- ‘Asal* 2013).^6^ In this dialogue, we clearly find the very rhetoric that Susan Sontag, in her seminal work on metaphors of cancer, had warned against: cancer is the epitome of “evil” and, as such, necessitates a relentless war (1991).

To answer the seemingly unanswerable question of why someone of Wahba’s good character could possibly get cancer, Neshan even wondered whether Wahba’s breast cancer was “a punishment from god” for taking off her headscarf in recent years. If Neshan later accredited the nature of his “difficult” questions to a mere rhetoric of provocation intended to highlight Wahba’s strength, his interview ultimately upheld existing ideas about cancer which directly or indirectly blamed the person with cancer for wrongdoing—in this instance moral and religious transgression (*Ana wal- ‘Asal* 2013). In spite or because of his overriding intention to locate Wahba’s cancer experience in the context of a narrative of perseverance and survival, the dialogue that unfolded transformed it into a narrative around sin and redemption.

It is also revealing that Neshan applauded Wahba for being an “icon and a hero” but still wanted to know how Wahba’s husband has been able to deal with her new female identity or rather the perceived lack of it: “I mean he loved your beauty, your femininity, and your aura (…) but with the mastectomy, the hair loss and everything (…).” At this point, Neshan’s voice trailed off into silence—as if there were some things that even this most provocative of interviewers felt must be left unsaid. In spite of Wahba’s efforts to present a “complete” image of womanhood—marked by a stylish wig, professional make-up, and a figure-hugging evening gown—both the presenter’s questions and even Wahba’s attempts to camouflage the signs of the treatment (and later her refusal to take off the wig) all pointed to the “lack” that she perhaps felt or has been made to feel (*Ana wal- ‘Asal* 2013).

Yet, for all its ambivalence, this public appearance of a woman with cancer attracted huge media attention in the Arab world. It bore witness to the larger cultural fact that certain archetypes of femininity are so deeply ingrained in Arab culture that many Arab women—unlike Wahba—have been condemned to live out their experience of illness, especially cancer, in silence.^7^ After the provocative line of questioning, the show was soon inundated with phone calls and emails in support of Wahba: Arab poets, journalists, pop stars, TV producers, as well as ordinary citizens hailed Wahba for the courage to speak openly about her illness and for inspiring hope in cancer patients. To a remarkable degree, however, Wahba’s public appearance inspired other female Arab public figures to speak out about their own cancer and to begin to forge a collective identity among women with experiences of this disease. In a phone call to the show, for example, the late Egyptian journalist and MBC presenter Fawzia Salama (who had been diagnosed with pancreatic cancer three months earlier) disclosed her own experience with cancer.

Perhaps one of the most powerful interventions, however, was that of the Lebanese TV and radio host Liliane Andraos who also, and for the first time, described her breast cancer diagnosis and treatment during the *Me and Honey* episode with Wahba. Speaking on the phone a week after she had finished her treatment, Andraos stressed that what had pained her most during her illness “was that my beloved left me because I had cancer. Why? Because I had lost my femininity? But I tell him (…) and tell any man that a woman’s femininity is not just in her physical appearance” (*Ana wal- ‘Asal* 2013). In many ways, Andraos’ sense of literal and figurative abandonment underscores one of the key tensions that Arab women confront when they are diagnosed with cancer, particularly breast cancer: the perceived assault on one’s identity as woman, wife, or lover.^8^

For Andraos, this “coming out” phone conversation on the episode was soon followed by a series of appearances on Lebanese radio and TV channels and newspapers to speak about her breast cancer experience. She would go on to become a regular guest on various Lebanese media platforms in support of any ongoing campaigns for breast cancer detection and/or to mark international cancer days. Her positive assertion that she “loved herself without hair” and that she now considers her illness a “blessing rather than wrath (*naqma*)” has been a challenge to the dominant discourse in an Arab world that largely sees cancer as infringing and disrupting, even destroying, a woman’s prospect of marriage and reproduction.^9^ If Andraos frequently narrated her cancer experience as one of “abandonment” by familial and social networks—“I forgive everyone who abandoned me during my health crisis” she declared—this sense of abandonment became a key driver for her public appearances (qtd. in Tohme 2015). In Andraos’ own words, “since my appearance on TV many women who were afraid to receive cancer treatment have been encouraged to do so.” Andraos goes on to proudly testify that she herself has spoken to many of these women convincing them “to return to their treatment” (Drive Time Show 2017).

In Wahba's (and Andraos’) public account of their breast cancer experience, we thus encounter a prime example of what Karen M. Kedrowski and Marilyn S. Sarow—in their study of a typology of breast and pancreatic cancer activism—identify as the role that grassroot survivors who draw on “their own experience as evidence” (2007, 43) play in facilitating “a sense of collective identity” that allow breast cancer patients and survivors to “assert themselves in the isolation of their illness” (55). It is also worth recalling here Potts’ assertion that first-person accounts which give “(…) access to intimate and very personal stories of another’s life” was one of the key building blocks for the development of the Women’s Health Movement of the 1970s and 1980s. As Potts contends, these accounts became “part of a conscious and radical determination to name and expose the socially constructed and punishable shamefulness of all aspects of female embodiment” (2000, 99). To anticipate the possible objection here that Wahba and her contemporaries’ fame grants them a social privilege and media access that would not be available to “normal” women, it is perhaps worth mentioning here that, a year after Wahba spoke about her diagnosis and treatment, she returned to another show *Wala Tehlam* [Do not Dream], also hosted by Neshan. This appearance was intended to mark the completion of her treatment. Perhaps most importantly, though, Wahba also came face to face with a female viewer (Elsi) who credited Wahba with helping her get an early cancer diagnosis and possibly saving her life: Elsi, who had undergone treatment for stage two breast cancer, told Wahba live on the show “if I hadn’t heard you, I wouldn’t have performed a mammography,” to which Wahba responded: “We are partners in illness and its journey and in our strength” (2014). In entering into this “partnership” with Elsi, Wahba transforms her individual experience of breast cancer into a collective one which broke the culture of silence surrounding female cancer.

## Yasmine Ghaith and the visual

In the case of Egyptian actress Yasmine Ghaith, we find that one of the defining features in the dissemination of her own breast cancer activism is not simply via verbal testimony but via visual media in the form of TV representations, photographs on social media, art works, and even public appearances that highlight the materiality of the body following a cancer diagnosis and treatment. To recall Ghaith’s breast cancer experience, her diagnosis came to the attention of audiences in Egypt and the rest of the Arab world through the successful TV drama series *Halawet al-Dunia* [The Sweetness of the World]. This series, which was an adaptation of its American counterpart *Chasing Life*, again aired in Ramadan of 2017 and featured some of the biggest names in the Arab acting industry.^10^ In *The Sweetness of the World*, a young professional woman called Amina (played by the renowned Hind Sabri) who is about to marry discovers she has leukemia and undergoes treatment. Yet one of the central mysteries in the drama for audiences concerned another character: who was the unknown actress who so powerfully plays the role of Hiba, a young woman with breast cancer that Amina meets at the hospital?

It would later emerge that Hiba was played by then-fashion designer and English teacher Yasmine Ghaith who was dramatizing her own breast cancer experience on screen. As the producer of the show Muhammad Mashish revealed, the series itself was inspired by Ghaith’s account of her illness on social media (“The Inspirational Cancer Survivor …” 2017). To make public her illness, Ghaith had posted details of her stage II breast cancer diagnosis on Facebook through a series of hashtags. Her story went viral and she was invited to participate in various interviews and awareness campaigns. However, the biggest and possibly the most dramatic development was the invitation to play the role of the breast cancer patient Hiba in the series. In spite, or because of the fact that she had no prior acting experience or training, Ghaith’s performance resonated with audiences for its realistic portrayal and for “the sensitive representation of an identifiable structure of feeling” (Ang 1985, 45).

To be clear, Ghaith’s participation in this dramatic rendition of her illness narrative challenged a number of long-standing cultural norms about how women should live this experience: Ghaith did not attempt to hide or disguise the physical signs of her treatment and especially the hair loss. She chose to shoot her first scene the day after a chemotherapy session, when she was still feeling the after-effects of the treatment, despite the production team’s desire to delay filming until she felt stronger. In a scene that—as Ghaith herself put it—“stunned all of Egypt” (2017), the young woman is seen defiantly taking off her wig to reveal her completely shaved hair and then laughing at the shocked reaction of the other characters.

For Ghaith, this performance of her illness narrative sprung from a resolve to (both literally and figuratively) be the protagonist of her own story and to speak back to a culture that has silenced cancer patients for so long. To resist an Arab culture that sees cancer “as equal to death” (2017), Ghaith (literally and figuratively again) refuses to be cast as, and play the role of a silent, voiceless victim of a death sentence. Her screen appearances confront the viewer with the very aspects of female cancer experience that had hitherto remained most repressed, namely, the all too visible physical changes to the body during treatment. As she makes clear in many interviews, Ghaith’s principal reason for participating in the television series was to challenge attitudes towards the physical appearance of people with cancer. In a comment on her appearance both in the series and in everyday life, she notes: “I wanted to change something in our culture which is the idea that we accept the appearance of a woman whatever it is (…)” (Wahed Min al-Nas 2018).

It is important to contextualize Ghaith’s act of defiance here by recalling that women across the Arab world historically comply with the aesthetic norm which sees a full head of hair as a signifier of beauty. After all, the Islamic veil or headscarf is frequently interpreted as an attempt to moderate what is regarded as a potentially erotic, tempting, and sexually tantalizing feature of a woman’s body. Yet, many secular and popular Arab cultural representations of women’s hair have reinforced and expressed this ideal. To recall, for example, the famous lyrics of Egyptian singer Abdel Halim Hafez in his song “Bitlumuni Leh?” [Why Do You Blame Me?] (1959):

I am the captive of my beloved, oh my weak heart.

I am trapped with the scent of her soft hair.

Which brushes against her cheeks, and then flows in the wind.

And everyone blames me and what can I do, oh my heart.

For the Lebanese novelist Abbas Beydoun, writing in his novel *Shahran li-Rula* [Two Months for Rula], a female character’s long and flowing hair is so sexually provocative that the young male protagonist masturbates while recollecting it (2018, 55). If women’s hair is deeply tied to a patriarchal ideal of femininity and sexuality, then any disruption to this ideal—such as hair loss or shaving because of cancer treatment—produces a cultural stigma where a woman who has cancer is deemed to be less than fully feminine. In a context where the vast majority of Arab women resort to some form of head covering when undergoing cancer treatment, whether it be a wig or a headscarf, Ghaith’s appearance both on screen and in public with a fully shaved head broke a major taboo.

In addition to her appearance in the TV series, Ghaith also became the ambassador for Baheya hospital which is regarded as the first hospital in Egypt specializing in the early—and entirely free—detection and treatment of breast cancer. She was also instrumental in setting up the first Egyptian support group (outside of the hospital framework) for cancer patients, their family members, and friends in 2019. To launch the support group, Ghaith announced in a Facebook post that support would take the form of conversations with Ghaith herself and/or group chats akin to the ones featured in *The Sweetness of the World* (Facebook 2019a)*.* By referring to the TV depiction of scenes where female cancer patients meet, and in indicating that the support group’s format was in response to the request of viewers, Ghaith clearly alludes to how these very viewers (and cancer patients especially) had identified with her character on screen to the point where they felt that they were part of the fictional world of Hiba and the real-life story of Ghaith. In many ways, this dialogic relationship between Ghaith and her audience has become one of the defining features of her breast cancer activism.

By persistently challenging the status quo of female cancer experience, Ghaith has now reached the position where many patients across the region see her as an inspiration. As she herself notes, “I suddenly found myself an ‘influencer’” and being described as “wonder woman.” She posts inspirational quotes and short diaries of her experience on social media regularly in order to overcome the pervading fear of cancer: “I got a disease that many people are scared of and I myself used to call it a bogeyman” only to then decide “to tell cancer ‘I love you’.” Her refusal, as she puts it, to “share some of the darkest” aspects of her cancer experience (such as the “unbearable” physical and mental pain) has throughout been driven by a determination to adopt a more positive attitude towards her illness journey which she credits with the “start of so many new things” including her acting career (2017). Of particular significance here are the ways in which Ghaith has built a community of patient followers through social media: Ghaith has documented her illness narrative and activism on digital platforms by “relating [her] individual perspectives to activist causes, organising activist communities, and negotiating shared realities with outsiders” (Greijdanus et al. 2020, 49). As Stefania Vicari and Franco Cappai remind us—in the context of their work on health activism and the digital media—“personal narratives of illness can connect online and, while certainly providing social support (…) to the members of a specific patient community, they can also add elements to health knowledge relevant to that community and to a wider public” (2016, 1667).

If Ghaith continues to advocate for breast cancer awareness and remains a key “motivational speaker” to those who are experiencing it, then her 10-piece jewelry collection that she released in 2019 can be seen as yet another attempt to record her cancer experience visually. Entitled “YG Pieces,” each of Ghaith’s jewelry pieces are made of 18ct handcrafted gold and what characterizes them is the mismatch and asymmetry of structure and form between and within each piece. The artist attempts to replicate, recreate, and capture the sutures and structures of the breast cancer wounds and scars following a mastectomy. Thus, each necklace, bracelet, ring, or earring is marked by an aesthetic of imperfection; Ghaith celebrates imperfection in the same way that she has decided to embrace her own “so-called” imperfect body following a cancer experience. Indeed, her favorite piece in the collection is the necklace “Scarlett,” whose name derived from the fact that “it looks exactly like my scar [from the surgery]. When I drew it, I knew this was my scar, and this was the trigger to the whole collection” (qtd. in Arab 2019).

In recreating her skin scar in jewelry, Ghaith effectively renders the impersonal surface of precious metal an intimate and personal index of her breast cancer wounds—whether psychological or physical. She also reinvents her cancer scar and the scars of others as an aesthetic object. As Lisa Cartwright puts it in the context of a discussion on visual media and the politics of breast cancer, Ghaith “reclaim[s] the scar as an object of aesthetic and political significance and, more profoundly, as an object of fascination, if not beauty” (1998, 128). However, this defiant stance against the invisibility and shame of cancer and breast cancer is further compounded through public appearances at high profile events during which Ghaith continues to render the story of her breast cancer experience visible. This is again undertaken through the topos of the scar and by repeatedly reconstituting the stigmatizing discourse associated with it. Insofar as the word “stigma” originates from the Greek stigma meaning “a mark made on the skin by pricking or branding” and which is ultimately intended as “a mark of disgrace” (*The Oxford English Dictionary*), then Ghaith’s engagement with the topos of the scar is an attempt to work through this intersection of surface wound and negative social perception: the permanent scar caused by her breast cancer surgery is redefined in each jewelry piece in new and empowering ways to the point where the scar itself is transformed into a site and symbol of perseverance against all odds.

To mark the launch of her collection in September 2019, Ghaith also appeared at the annual el-Gouna Film Festival dressed in a white T-shirt that read “our scars are our pride” while wearing a necklace and earrings from her scar jewelry collection. For Ghaith, as she posted on social media, “I wanted to tell anyone who is embarrassed of their scars and imperfections and who try to disguise it that it is an honour not a disgrace (…) It is something to be proud of” (2019b). While at the same festival, she appeared at another red-carpet event in a white evening dress whose upper body was specifically designed so that her necklace “Scarlett” fell directly over her right breast where the surgery scar was located. In all this, what we encounter are the ways in which the visible scar on the skin and the scar etched on the jewelry piece interact in such a way as to invoke a haptic visuality that speaks of trauma, healing, and acts of resistance. In short, the object/necklace interacts with the history of the wound etched on the skin and it is precisely because of, and through, this intimate connection that the viewer is given an entry point to witness the tactile and visual dimension of Ghaith’s embodied breast cancer experience.

Finally, Ghaith’s participation in the TV series, her public and visual exposure of her cancer experience as well as her jewelry collection are all marked by what Rosemarie Garland-Thomson calls “visual activism”: Ghaith has positioned herself in the “public eye, saying ‘look at me’ instead of don’t stare” (2009, 193). To perform her breast cancer in this way, she both attracts and defies the gaze of the viewer and unsettles social and cultural perceptions of the disease. If, as Alan Radley and Susan E. Bell note, “making breast cancer public involves making breast cancer visible, something designed to breach the cultural cloak that has lain over both cancer generally and breast cancer in particular” (2007, 368), then Ghaith’s challenging of the viewer’s gaze reconstitutes an act or a series of acts of resistance against the taboo of breast cancer. In rendering the images of herself with cancer public and visible, Ghaith ultimately performs a form of activism that seeks to unsettle how people perceive, think, and even view a woman with breast cancer.

## Namaa al-Ward and performativity

In a play entitled *Ana fi Intizarak* [I am Waiting for You] (2017), written by myself and directed by the Lebanese feminist Lina Abyad, the Iraqi actress Namaa al-Ward shaves her hair and, more significantly, bears her surgically reconstructed breast on stage for the first time in an Arab theatre.^11^ Al-Ward performs the story of Ilham, an Iraqi woman with breast cancer whose story is brought into dialogue with a female literary figure by the same name from the novel *Ghayeb* [*Absent*] (2004) by Iraqi Betool Khedairi.^12^ She thus plays the role of two women, one fictional and one real, to create a dialogic relationship between the lived experience of breast cancer and its (often problematic) representation in Arabic literature.^13^ Yet al-Ward’s participation added another layer to the portrayal of the testimonies on stage because the actress herself had experienced breast cancer and her commitment to the role was throughout driven by activism: “I want people to know that they can survive cancer! I want them to see me and know that I am here. That I made it,” she would repeat throughout the rehearsals. In order to supplement the testimonies on stage with her own story, al-Ward would break not only one but two cancer taboos: she shaved her hair on stage and revealed her surgically reconstructed breast.

To begin with the act of hair shaving, on the opening night of *I am Waiting for You*, the audience is invited to enter the historic Madina Theatre in Beirut, Lebanon, while al-Ward sits in the center of the stage. As the audience take their seat, the lights are dimmed and a spotlight is directed onto the figure of al-Ward. Next, the Spanish film director, Paloma Yañez Serrano—who is in Beirut to film a documentary on the performance—steps on stage with a pair of scissors and a razor. Finally, Serrano slowly and meticulously cuts al-Ward’s hair with the scissors before shaving her head until she is completely hairless. In this whole process—which takes more than ten long minutes and provokes visible and audible discomfort or embarrassment among some audience members—al-Ward smiles and stares defiantly at the audience before finally walking out of the stage (al-Ward 2017).

Al-Ward’s decision to shave her hair on the opening night staged the symbolic female rite of passage from one state to another, namely, to cancer patient. It is well documented that many women preparing to undergo chemotherapy pre-emptively cut their hair before it begins to fall out but this act is normally performed privately. Accordingly, al-Ward’s public shaving of her hair was an act of resistance against what we have already seen to be prevailing norms around the social and symbolic function of female hair. If, as Margo Maine argues, “hair is never hair. It has consistently served as a second nonverbal language to express critical sentiments, telling the story of women’s lives like nothing else” (2018, 122), then the significance of al-Ward’s performative act is that it confronts audiences with the vulnerability of the female body with cancer, pointing to those aspects of bodily changes that have often brought shame to those diagnosed with the disease. Yet, al-Ward also subverts these deeply ingrained cultural norms from the outset. Her control over the representation of her bodily account of breast cancer reflects a determination to have agency over her body and a wish to empower other women who, like her, have experienced cancer. However, her act of defiance triggered visceral reactions from the audience on the opening night: A prominent male critic sitting in the front row averted his gaze from the stage, while another male audience member was heard angrily exclaiming: “What is this nonsense?” This apparent male discomfort is in stark opposition to the reaction of female audience members who experienced al-Ward’s act as a centrifugal point of connectivity and solidarity: A female audience member cried silently, while another mouthed a silent “Thank you” to al-Ward as she stepped from the stage with her shaven hair. In the aftermath of the first performance, other female audience members embraced al-Ward and posted supportive messages on social media: “What courage and strength from her part [al-Ward] … in a country that always wants to cover it up, deny it” (Hamouche 2017).

In many ways, al-Ward’s display of her reconstructed breast and her graphic description of the surgical procedure she underwent after diagnosis was her single most disruptive act of breast cancer activism. As Emilia Nielsen writes “disruptive breast cancer narratives have the potential to change public perceptions of the disease,” adding that “disruptive breast cancer narratives in public circulation trouble the cultural politics of breast cancer and in doing so work to write a new history of the breast cancer experience” (2019 145). To recall this moment that takes place half-way through her performance, al-Ward draws the audience’s gaze to the points on her right breast where the incisions were made, then tells them how the breast was patched together, and the nipple reconstructed: “Look how beautiful it is. Better than the old one!” By exposing the acute bodily manifestations of her cancer experience in this way, al-Ward refuses the dominant patriarchal view that a breast cancer diagnosis is the demise of female sexuality and instead—like Ghaith before her—insists upon the “beauty” of her post-cancer breast. For Rebecca Schneider, writing on the significance of the “explicit body in performance,” this form of theatre “is foremost a site of social markings, physical parts and gestural signatures of gender, race, class, age, sexuality – all of which bear ghosts of historical meaning, markings delineating social hierarchies of privilege and disprivilege” (1997, 2). If women’s literary narratives of cancer are read in private spaces, DeShazer reminds us, then theatre’s potential to disrupt how we respond and interact with cancer accounts “evokes reciprocity and foster[s] activism”: “Theatre offers a public space in which audiences can reckon with the physical and emotional ravages as well as the politics of the disease” (2010, 53). In forcing her audience to bear witness to the corporeal “markings” of her cancer experience, al-Ward not only challenges the historical exclusion of female bodies with breast cancer from the Arab stage but the socio-political invisibilization of such bodies from the stage of Arab history.^14^

## Elissa and sonic stories

In early August 2018, the Lebanese pop-singer Elissa, who is widely known across the Middle East, released a new song and accompanying music video https://www.newarab.com/news/lebanon-pop-star-elissa-reveals-cancer-battle-music-video entitled “Ila Kol Elli Bihibbouni” [To All Those Who Love Me]. It garnered over 8 million views on social media within a few weeks of its release.^15^ If Elissa’s music videos are always highly anticipated events, the unprecedented interest in this specific video was for different reasons: the new video revealed Elissa’s breast cancer diagnosis and described her treatment journey. In “To All Those Who Love Me,” Elissa was effectively writing, singing, and performing a “coming out” cancer narrative.

To briefly reconstruct the context, Elissa—whose original name is Elissar Zakaria Khoury—was first diagnosed with breast cancer in December 2017. However, the singer kept her diagnosis totally secret and did not even inform close family members like her mother and sister. As she underwent treatment, she continued to perform concerts and even collapsed and lost consciousness on stage while performing live at a concert in Dubai in February, 2018. When a recording of the singer’s collapse went viral and fans expressed concerns, she tweeted: “Thank you for all your well wishes! I love to keep my Dubai concerts memorable. Jokes aside, I’m healthy thank you for ur support,” adding: “I am feeling better. Nothing serious don’t worry!” (qtd. in Mekky 2018). In August 2018 and after the completion of her treatment, Elissa released what has since been described in the media as her taboo breaking video (“Elissa Breaks Taboo …” 2018).

For Elissa, “To Everyone Who Loves Me” is a kind of documentary in music video form. It chronicles Elissa’s illness journey from the moment of diagnosis to the completion of her treatment using both real footage and dramatic reconstructions as well as voice notes from the singer herself. Firstly, the video depicts Elissa inside an MRI and we hear an ominous voice informing her that she has early stages of breast cancer. Following this scene, we hear a voice recording of Elissa herself talking to a female friend about her illness and wondering whether she will be able to see “all the people I love again. Those who love me back (….): There is this moment when you think that’s it,” she declares, “You won’t exist tomorrow.” Her voiceover goes on to detail the singer’s everyday life as she balances a secret course of treatment with her career as a singer in the public eye:

I do radiotherapy. I go to the studio. I finish another session. I rest for two hours. I go to the studio again. Once a week I go live on TV. Sometimes it’s not me I feel sorry for. Sometimes you feel sorry for the people you truly love ... This song is for all the people we love. And I love you so much.

The song’s lyrics and accompanying images correspondingly juxtapose Elissa in the hospital, with her doctors, undergoing treatment, and caring for her breast wound with Elissa in the studio, preparing for a concert, on stage performing, including a recreation of her collapse at the concert in Dubai. In the final part of the music video, we see Elissa walking silently on stage in a glamorous evening gown while a silent message appears on screen: “I’ve recovered. I’ve beaten the illness and I won … Early detection of breast cancer can save your life … Don’t ignore it, face it. Do it not only for yourself, but for your loved ones” (Elissa 2018).

If Elissa’s “To Everyone Who Loves Me” is a “coming-out” narrative, what is particularly interesting is that it did not appear at the point of diagnosis but only after the successful completion of her treatment. Its retrospective temporality bears witness once again to the prevailing culture of silence that surrounds the lived experience of female cancer in the present. As Elissa depicts herself covering up her cancer in the video and pretending that she is perfectly well, she dramatizes one of the key tropes or experiences that many Arab women resort to following a cancer diagnosis, namely, “passing” or “the way people conceal their impairments to avoid the stigma of disability and pass as ‘normal’” (Brune et al. 2013, 1). To be clear, Elissa herself admits that she felt compelled to keep her own diagnosis a secret out of a fear of being stigmatized—“I didn’t want them to look at me and say ‘what a pity, she is ill’” (qtd. in Ahmed 2021)—but her music video powerfully dramatizes this fear itself: Its subject is not so much “cancer” as the culture of (self)censorship that produces such phenomena as “passing.” For Elissa, “To All Those Who Love Me” is a plea for sympathy and understanding not only from those who may be inclined to judge or condemn her as a woman who has had cancer but from those who may feel that her decision not to disclose her illness broke or betrayed their trust in her.

In her final written message that appears on screen, Elissa positions herself as an activist advocating for early screening and detection and she has subsequently participated in many campaigns for breast cancer awareness: she was, for example, ambassador for the national breast cancer campaign in Egypt in 2018 to support breast screenings. As part of her involvement in the Egypt campaign, the singer performed a concert on 19 January 2019 and the yield from this event was donated to Egyptian breast cancer patients. In February 2019, she appeared at the Dubai Health Forum and shared candid details about her lived experience of breast cancer with the crowd present. In October 2021, and in response to the economic crisis in Lebanon, the singer announced an initiative to financially support women in the country to undergo breast cancer screenings. As part of this initiative, she also launched the campaign “Together We Fight Breast Cancer” which was undertaken in collaboration with Rizk Hospital in Beirut and a number of associations. The campaign aimed “to conduct 10,000 x-ray examinations in all Lebanese regions” including in towns and villages in the North and South of the country (Sadler 2021).

To sum up, “To Everyone Who Loves Me” represents a new multimodal form of female cancer activism in the Arab world which combines music, images, text, and voiceovers. The music video has now been viewed over 27 million times on social media and has become a model for subsequent cancer awareness campaigns. At the same time, Elissa’s status as a cancer survivor and activist has not diminished or detracted from her celebrity but has become part of her global brand. If many women who are diagnosed with cancer ask “Why me?”, she says she never asked that question until “after the video clip dropped and after the response it received” and her answer was a positive one. In that moment, she recalls: “I realised that God picked me because I am an influential person and I have a voice that is heard” (qtd. in Badih 2018). Elissa’s interpretation of her own experience here sees it as a divine intervention that is intended to propel her towards an activist path. This activism, as we have seen, is to raise awareness of the disease and to encourage early screening and treatment “particularly given ‘the high incidence rates’ of breast cancer in the region” (Saseendran 2024).^16^

## Conclusion

We have seen how the accounts of these various female artists and media figures employ the arts and popular culture to tell their accounts of breast cancer. From media stories to visual, sonic, and performative practices, the forms and genres they draw on open up new spaces and platforms through which they can tell of their individual experiences in such a way as to reach a mass audience and to engage ordinary citizens in affective and intense ways. If their accounts chronicle individual experiences, they nevertheless invoke a collective sense of shared vulnerability that is both physical and social. In this sense, they overcome the difference between the individual and the collective by always locating the individual story within a collective.

To read these very different artistic and popular productions, which range generically from realism to melodrama, as a collective body of work, we also see how they blur the boundary between art and activism, representation and action. By bringing to light “shared conditions of vulnerability” with others who have experienced the disease, these public figures “expose frameworks of inequality” and “forms of interconnectedness” that Arab women with cancer experience. In breaking the silence surrounding women’s experiences of cancer, they also directly and indirectly “produce and transform the emotions that create solidarity and strengthen participation” in collective action (Klawiter 2000: 69). This increased sense of a shared reality also blurs national boundaries and identities, facilitating Arab women’s regional interconnectedness and solidarity around the experience of cancer. This, in turn, broadens the scope of their activist campaigns and initiatives as well as its potential for yielding change across the region.

For Jessica Stites Mor and Maria del Carmen Suescun Pozas, writing on the art of solidarity, “a large part of political action is derived from a sense of perceived belonging, a carefully articulated worldview within which one can identify oneself as a member of a social group” (2018, 5). To focus more narrowly on cancer activism, Klawiter also argues that certain social movements (including breast cancer movements) “achieve their greatest impact in the cultural arena, through changing popular images, ideas, emotions and identities” (2004, 851). By bringing breast cancer accounts into popular media like television and music video clips, the women discussed in this essay not only change the media representation of cancer but the attitude of the mass of people who consume popular media.

In many ways, of course, I think it is too early to speak of or document a “paradigm shift” in the breast cancer regime in the Arab world but what I have called art and popular breast cancer activism can be seen as an ongoing collective attempt to challenge public discourses around Arab women’s cancer. To conclude, the women featured in this essay contribute to an *unfolding* breast cancer activist movement that is rooted in survivors’ accounts and that is not solely reliant on the work of breast cancer foundations and charities. In their ongoing work, we witness how Arab women who have experienced cancer are thus generating a new feminist body politics that challenges the traditional status quo of ill female bodies and subjectivities.

## Data Availability

The author confirms that the data supporting the findings of this study are available within the article.

## Declarations

### Endnotes

^1^ I use a general system of translation and only use the ayn (‘) and hamza (’). I also present all Arabic names and titles of people, places, and texts in the form most familiar to an English-speaking audience and/or the form used by the writers and the works under discussion.

^2^ In 2007, al-Amoudi won the first International Woman of Courage Award which is presented by the US Department of State.

^3^ One such organization is the Barbara Nassar Organization in Lebanon.

^4^ For a study of Banna’s breast cancer account, see Hamdar (2020).

^5^ These include the Lebanese Breast Cancer Foundation, Baheya Foundation in Egypt, The Zahra Breast Cancer Association in Saudi Arabia, Think Pink in Bahrain, Best Friends and Pink Caravan in the UAE.

^6^ Unless otherwise stated, all translations from the Arabic are my own.

^7^ For studies of breast cancer in Arab contexts, see Fearon et al. (2020).

^8^ See, for example, Hamdar (2021).

^9^ See Fearon et al. (2020).

^10^ These include Hind Sabri and the late Ragaa al-Geddawi.

^11^ The play is an ethnodrama based on interviews with female cancer patients from across the Arab world.

^12^ The character Ilham in Khedairi’s *Absent* and the Iraqi woman whose testimony features in the play both experience their cancer as “a disease that emerged out of the body politic of their nation.” The dialogism established between the real and the fictional is intended to “draw out this symbolic resonance” (Hamdar 2021, 203).

^13^ See, for example, Hamdar (2019) on illness and disability representations in Arabic literature.

^14^ As far as everyone is aware, this is the first time a female breast is exposed so explicitly on the Arab stage and it is the breast of a woman who had experienced breast cancer.

^15^ In 2005, 2006 and 2010, she received the World Music Award for Best Selling Middle Eastern Artist, and became the first Lebanese singer to receive the World Music Award for Best Selling Middle Eastern Artist. https://www.egypttoday.com/Article/4/67784/Elissa-to-perform-in-Dubai-on-Apr-5.

^16^ For breast cancer rates see, for example, el-Saghir et al. (2007), Ezzat et al. (1999), and Fearon et al. (2020).
