# Correction: Breaking Taboos: Arab Breast Cancer Activism in Art and Popular Culture

**DOI:** 10.1007/s10912-024-09900-x

**Published:** 2024-09-26

**Authors:** Abir Hamdar

**Affiliations:** School of Modern Languages and Cultures, Durham University, New Elvet, Elvet Riverside, Durham, DH1 3JT UK

**Correction: Journal of Medical Humanities**

10.1007/s10912-024-09886-6

In the recently published article, the third paragraph in the “Introduction” section is repeated. This should be removed.

The original article has been corrected.

